# Mental Health and Gender Affirmation of Black and Latine Transgender/Nonbinary Youth Compared to White Peers Prior to Hormone Initiation

**DOI:** 10.1016/j.jadohealth.2023.06.022

**Published:** 2023-08-23

**Authors:** Stanley R. Vance, Diane Chen, Robert Garofalo, David V. Glidden, Diane Ehrensaft, Marco Hidalgo, Amy Tishelman, Stephen M. Rosenthal, Yee-Ming Chan, Johanna Olson-Kennedy, Jae Sevelius

**Affiliations:** a Child and Adolescent Gender Center, Division of Adolescent and Young Adult Medicine, Department of Pediatrics, Benioff Children’s Hospital, University of California, San Francisco, San Francisco, California; b Gender and Sex Development Program, Potoscnak Family Division of Adolescent and Young Adult Medicine, and Pritzker Department of Psychiatry and Behavioral Health, Ann and Robert H. Lurie’s Children’s Hospital, Chicago, Illinois; c Department of Psychiatry and Behavioral Sciences, and Pediatrics, Northwestern University Feinberg School of Medicine, Chicago, Illinois; d Gender and Sex Development Program, Potoscnak Family Division of Adolescent and Young Adult Medicine, Department of Pediatrics, Ann and Robert H. Lurie’s Children’s Hospital, Northwestern University Feinberg School of Medicine, Chicago, Illinois; e Department of Epidemiology and Biostatistics, University of California, San Francisco, San Francisco, California; f Child and Adolescent Gender Center, Division of Pediatric Endocrinology, Department of Pediatrics, Benioff Children’s Hospital, University of California, San Francisco, San Francisco, California; g Gender Health Program, Medicine-Pediatrics Division, General Internal Medicine and Health Services Research, Department of Medicine, David Geffen School of Medicine at University of California, Los Angeles, Los Angeles, California; h Department of Psychology and Neuroscience, Boston College, Chestnut Hill, Massachusetts; i Child and Adolescent Gender Center, Division of Pediatric Endocrinology, Department of Pediatrics, Benioff Children’s Hospital, University of California, San Francisco, San Francisco, California; j Division of Endocrinology, Department of Pediatrics, Boston Children’s Hospital, Harvard Medical School, Boston, Massachusetts; k Center for Transyouth Health and Development, Division of Adolescent and Young Adult Medicine, Department of Pediatrics, Children’s Hospital Los Angeles, Keck School of Medicine, University of Southern California, Los Angeles, California; l Department of Medicine, Center for AIDS Prevention Studies, University of California, San Francisco, California

**Keywords:** Transgender persons, Mental health, Gender affirmation

## Abstract

**Purpose:**

To compare baseline mental health symptoms and gender affirmation between Black/Latine versus White transgender/nonbinary youth (BLTY vs. WTY) and examine relationships between gender affirmation and mental health symptoms, and whether associations differed by race/ethnicity subgroup.

**Methods:**

Baseline data were analyzed from the gender-affirming hormone cohort of the Trans Youth Care United States Study—a 4-clinic site, observational study. Mental health symptoms assessed included depression, suicidality, and anxiety. Gender affirmation measures included the parental acceptance subscale from the perceived Parental Attitudes of Gender Expansiveness Scale-Youth Report; non-affirmation, internalized transphobia, and community connectedness subscales from the Gender Minority Stress and Resilience Measure—Adolescent; and self-reported living full time in affirmed gender. Fisher exact tests and independent sample *t* tests compared mental health symptoms and gender affirmation between subgroups. Logistic regression analyses evaluated associations between gender affirmation and mental health symptoms. Interaction analyses assessed differences in associations between subgroups.

**Results:**

The sample (mean age 16 years, range 12—20 years) included 92 BLTY (35%) and 170 WTY (65%). Subgroups had comparable prevalence of depression and anxiety symptoms. WTY had higher prevalence of lifetime suicidality (73% vs. 59%; *p* = .02). There were no differences in gender affirmation. Among the whole sample, higher parental acceptance decreased odds of depression symptoms. Not living in affirmed gender increased odds of depression symptoms. Higher non-affirmation and internalized transphobia increased odds of depression and anxiety symptoms and suicidality. Associations did not vary by subgroup.

**Discussion:**

BLTY and WTY had comparable mental health symptoms. For both subgroups, gender affirmation decreased odds of those symptoms.

Black and Latine transgender/nonbinary youth (BLTY) share minoritized experiences of both belonging to a racial/ethnic minority and being gender diverse. While Black and Latine gender-diverse individuals have culturally heterogenous experiences, both face intersecting forms of stigma and discrimination, particularly transphobia and racial/ethnic discrimination [1]. Experiences of stigma can interact, synergistically contributing to poor health outcomes and health inequities [2]. Prior studies of Black and Latine gender-diverse adults, both as a combined cohort and separately, demonstrate that they experience high burdens of violence, socioeconomic disadvantages, mental health symptoms, and poor health outcomes [3–5]. Moreover, studies comparing Black and Latine gender-diverse adults to White gender-diverse adults have found that Black and Latine gender-diverse adults experience higher rates of mental health symptoms and victimization than White gender-diverse adult peers [6–8]. These health inequities must be understood within the context of systemic racism and transphobia faced by Black and Latine gender-diverse adults [3,6,9]. Unfortunately, a paucity of studies explore whether BLTY experience the marked psychosocial inequities reported by Black and Latine gender-diverse adults. Empirically assessing whether BLTY experience health inequities related to psychosocial functioning is critically important for supporting Black and Latine gender-diverse people throughout their life course.

Despite limited data focused on BLTY, literature suggests that they may be vulnerable to mental health inequities based on studies of the minoritized groups to which they belong: (1) gender diverse and (2) Black and Latine. Gender-diverse youth experience higher rates of mood disorders and suicidality compared to cisgender peers [10–13]. Moreover, Black and Latine youth populations have experienced a recent, substantial increase in suicide death rates compared to White youth [14,15]. Black and Latine youth also have high rates of mental health concerns including depression, anxiety, and substance abuse [16,17] with racial discrimination being a significant risk factor [18]. A study examining mental health symptoms in a school-based sample of BLTY found high prevalence of depression symptoms and suicidality, and race-based harassment was one risk factor [13]. However, most quantitative studies of clinical samples of youth presenting for gender-affirming medical care have not examined mental health symptoms specifically among BLTY or compared them with White transgender/nonbinary youth (WTY) [11,19–23].

Social-contextual factors that may mitigate mental health risk among BLTY have not been investigated. Early research suggests that gender affirmation, which refers to an interactive and interpersonal process by which gender-diverse people receive social recognition and support for their gender, may mitigate mental health risk among gender-diverse youth [24–27]. The Model of Gender Affirmation is a framework that posits that increased access to gender affirmation can improve health outcomes among gender-diverse people [24]. For example, gender affirmation via parental support of youth’s gender diversity is associated with lower levels of depression and suicidality [25,26]. Community connectedness, another form of gender affirmation where individuals with shared identities come together to support and affirm one another, is also important to the mental health of gender-diverse youth [28]. Alternatively, gender non-affirmation in the form of internalized transphobia–that is, the internalization of stigmatizing social messages regarding gender identity and/or expression–is associated with depression and anxiety among gender-diverse youth [29]. However, studies to date lack a specific focus on BLTY, their experiences with gender affirmation, and how their experiences compare with WTY. Prior studies have also not explored how BLTY’s experiences with gender affirmation influence their risk for or are protective against mental health symptoms.

In 2015, four pediatric gender clinics at United States academic medical centers were funded by the National Institutes of Health to study the medical and psychosocial impact of gender-affirming medical treatment for youth with gender dysphoria [30]. These centers formed the Trans Youth Care United States (TYCUS) Study composed of Children’s Hospital Los Angeles/University of Southern California, Ann and Robert H. Lurie Children’s Hospital of Chicago/Northwestern University, Boston Children’s Hospital/Harvard Medical School, and Benioff Children’s Hospital/University of California, San Francisco. As a multisite, longitudinal observational study, the TYCUS Study evaluates the mental and physical health of gender-diverse youth initiating gender-affirming medical treatment, and therefore, affords an opportunity to explore mental health and gender affirmation among subsamples of BLTY and WTY.

To address gaps in the existing literature, this study assessed whether disparities described between Black and Latine gender-diverse adults related to mental health symptoms (e.g., depression, lifetime suicidality, and anxiety) and experiences with gender affirmation (e.g., living full time in the affirmed gender, parental acceptance, internalized transphobia, non-affirmation, and community connectedness) were also present in the TYCUS sample. We hypothesized that BLTY would have higher rates of mental health symptoms and lower levels of gender affirmation compared to WTY. Additionally, we examined associations between mental health symptoms and gender affirmation, exploring differences between BLTY and WTY. We hypothesized that lower levels of gender affirmation would be associated with more mental health symptoms.

## Methods

### Participants and recruitment

The current study uses baseline data from a subsample of the TYCUS gender-affirming hormone cohort, which is comprised of youth initiating gender-affirming hormones in the forms of testosterone or estrogen for phenotypic transition. Youth were recruited from July 2016 through June 2019. The inclusion criteria included: (1) presence of gender dysphoria based on clinician evaluation, (2) appropriateness for initiation of medical treatment as determined by the primary clinical team, (3) English proficiency, and (4) accessing clinical services at one of the study sites. The current subsample excludes youth with prior pubertal suppression as these youth had in some cases years of medical gender affirmation compared to youth initiating gender-affirming hormones without prior pubertal suppression. Prior research has demonstrated youth presenting for pubertal suppression have better psychosocial functioning compared to youth presenting for gender-affirming hormones [22]. The age range for this subsample is 12–20 years. Other publications have more comprehensively detailed the TYCUS study methods [22,30]. Study procedures were approved by institutional review boards at all study sites.

### Measures

#### Demographics.

Youth reported age, gender identity, sex designated at birth, race, and ethnicity. For gender identity, participants were asked, “How do you currently identify in terms of your affirmed gender?” They selected from eight response options or indicated “other” and specified. Responses were recoded into the following categories: transfeminine, transmasculine, and nonbinary. Participants were asked to check all race/ethnicity categories that apply to them from the following: Black, Hispanic or Latino, White, American Indian/Alaska Native, Asian, Native Hawaiian or other Pacific Islander, or other. For this study, youth who identified as Black and/or Hispanic/Latine were categorized as BLTY, including youth who selected multiple race/ethnicity categories that included Black and/or Latine as one of the categories. Youth who identified as White and no other category were categorized as WTY. Youth who were not categorized as BLTY or WTY were excluded from this analysis because our focus was on the experiences of BLTY whose adult counterparts have described a higher burden of mental health inequities than their White peers [3–5].

#### Mental health symptoms.

Youth completed the 21-item Beck Depression Inventory II to assess presence and severity of depression symptoms. Items are rated on 4-point scale and summed for a total score. For participants with missing items but with ≥75% completed, their total score was imputed with the participant’s mean of completed items; four participants with <75% completed subscale items were excluded from the analyses using this measure. Total scores were compared to standard cutoffs for minimal (0—13), mild (14—19), moderate (20—28), and severe depression (29—63) [31]. For this study, scores were dichotomized to minimal depression symptoms and mild-to-severe depression symptoms. Youth were also queried via a yes/no question for lifetime suicidality with the following question: “Have you ever thought about killing yourself?” *Youth completed the Revised Children’s Manifest Anxiety Scale, Second Edition* with 49 yes/no items. “Yes” responses were summed and transformed into total anxiety *T* scores. *T* scores > 60 are considered clinically significant anxiety symptoms; a dichotomized variable was created to indicate presence or absence of clinical symptoms [32].

#### Experiences of gender affirmation.

Youth responded yes/no to the following: “Are you living full time as your affirmed gender now?” Parental support was measured by a 6-item subscale of the Perceived Parental Acceptance Subscale from the Parental Attitudes of Gender Expansiveness Scale-Youth Report [33]. Using a 5-point Likert scale (1 = strong disagree, 5 = strongly agree), this subscale measures youth’s perceptions of whether parents are proud of them, allow them to be themselves, advocate for their rights as a gender-diverse person, protect them against gender-based prejudice, and support their phenotypic gender affirmation (Cronbach alpha = 0.86). Subscale responses were summed. Non-affirmation, internalized transphobia, and community connectedness were measured by the Gender Minority Stress and Resilience Measure—Adolescent that contains items rated on 5-point Likert scales (1 = strongly disagree to 5 = strongly agree) [28]. The Non-Affirmation subscale contains six items (Cronbach alpha = 0.88); an example item is, “I have to repeatedly explain my gender identity to people or correct the pronouns people use.” The internalized transphobia subscale contains eight items (Cronbach alpha = 0.90); an example item is, “I resent my gender identity or expression.” The community connectedness subscale contains five items (Cronbach alpha = 0.79); an example item is, “I feel part of a community of people who share my gender identity.” Higher subscale scores indicate higher levels of parental acceptance, non-affirmation, internal transphobia, and community connectedness. For participants with missing subscale items but with ≥75% of items completed, their total score was imputed with the participant’s mean of completed items; participants with <75% completed subscale items were excluded from analyses of these measures (parental acceptance (n = 7), non-affirmation (n = 4), internalized transphobia (n = 3), and community connectedness (n = 5)).

#### Statistical analyses.

Descriptive and multivariable analyses were conducted in STATA 17.1 (StataCorp, College Station, Texas). Descriptive analyses were conducted for demographics, mental health symptoms, and gender affirmation measures. Medians and interquartile ranges were used for continuous variables; frequencies and percentages were used to summarize categorical variables. Differences in demographics by designated sex at birth were assessed via Fisher exact tests. Independent sample *t* tests and Fisher exact tests were used to assess differences in continuous and categorical variables, respectively. Differences in mental health symptoms and gender affirmation measures between BLTY and WTY were evaluated using Fisher exact tests and independent sample *t* tests. Logistic regression analyses were used to model each mental health symptom as a function of a gender affirmation measure while adjusting for age as a continuous variable and designated sex at birth, given associations of these covariates with mental health symptoms [34,35]. These analyses were conducted for the entire sample. Five logistic regression models were run for each mental health symptom for a total of 15 models. Additional logistic regression analyses were conducted to determine if the associations of experiences of gender affirmation with mental health symptoms varied by race/ethnicity subgroup. For these analyses, each mental health symptom was modeled as a function of one experience of gender affirmation, race/ethnicity subgroup, a term for interaction between the experience of gender affirmation and race/ethnicity subgroup, and demographic covariates. As an example, one analysis includes depression symptoms as the outcome with parental acceptance versus race/ethnicity subgroup interaction term, parental acceptance term, race/ethnicity term, and demographic covariates. Five models were run for each mental health symptoms for a total of 15 models. Logistic regression analyses were corrected for multiple comparisons using the Benjamini—Hochberg method with an overall false discovery rate of 0.05. Cases with missing data were list-wise deleted for all analyses.

## Results

A total of 315 youth completed baseline assessments. Data from 262 youth were included in our analyses; 24 youth were excluded due to a prior history of pubertal suppression and 29 youth were excluded for not being categorized as either BLTY or WTY. Table 1 presents demographic data for the analytic sample. The majority were designated female at birth (68%) and transmasculine (63%). Of the BLTY, 85% were Hispanic/Latine and 20% were Black with 4% identifying as both Hispanic/Latine and Black. There were no significant differences between BLTY and WTY in designated sex at birth or age; median age was 16 years for both subgroups (*p* = .83).

Table 2 summarizes the prevalence of mental health symptoms for the BLTY and WTY subgroups. Comparable proportions of BLTY and WTY endorsed elevated depression symptoms (48% vs. 57%, *p* = .19) and clinically significant anxiety symptoms (57% vs. 60%, *p* = .59). Fewer BLTY had lifetime suicidal ideation than WTY (58% vs. 73%, *p* = .02). Experiences of gender affirmation are summarized in Table 3. The majority of BLTY (81%) and WTY (79%) were living full time in their affirmed gender (*p* = .75). When comparing subgroups, there were no significant differences in parental acceptance, non-affirmation, internalized transphobia, or community connectedness.

Multivariate logistic regression models assessing associations between mental health symptoms and gender affirmation for all youth are depicted in Table 4. Higher levels of parental acceptance were associated with lower odds of depression symptoms (adjusted odds ratio (aOR), 0.91; 95% CI, 0.87—0.96). Not living in the affirmed gender was associated with higher odds of depression (aOR, 2.46; 95% CI, 1.21—4.88). Higher levels of nonaffirmation were associated with higher odds of depression symptoms (aOR, 1.08; 95% CI, 1.03–1.13), lifetime suicidal ideation (aOR, 1.10; 95% CI, 1.04—1.15), and anxiety symptoms (aOR, 1.11; 95% CI 1.05–1.17). Higher levels of internalized transphobia were also associated with higher odds of depression symptoms (aOR, 1.10,95% CI 1.07—1.14), lifetime suicidal ideation (aOR, 1.04; 95% CI 1.01—1.08), and anxiety symptoms (aOR, 1.10; 95% CI 1.06—1.14).

Additional logistic regression analyses were conducted to explore whether interactions between each experience of gender affirmation and race/ethnicity subgroup were associated with mental health symptoms (Table 4). Gender affirmation by subgroup interactions was not statistically significant.

## Discussion

Baseline data from an observational study of transgender/nonbinary youth recruited from pediatrics gender clinics were analyzed to investigate mental health symptoms and experiences with gender affirmation among BLTY compared with WTY prior to initiating gender-affirming hormones. There were no differences in rates of elevated depression and anxiety symptoms between BLTY and WTY. Furthermore, BLTY actually had lower rates of lifetime suicidality compared to WTY. Moreover, compared to WTY, BLTY had similar levels of gender affirmation. For all youth in the sample, lower levels of gender affirmation were associated with higher mental health symptoms, and these associations appear to be similar when comparing race/ethnicity subgroups.

Contrary to hypotheses, BLTY and WTY had comparable rates of depression and anxiety symptoms as well as experiences of gender affirmation. Our hypothesis was based on well-documented mental health and psychosocial disparities experienced by Black and Latine gender-diverse adults when compared to their White counterparts [6–8]. There are several possible explanations for these findings. An important possibility is that the current study employs a clinic-based sample of youth who by definition have caregivers, who are sufficiently supportive to bring them to multidisciplinary pediatric gender centers and consent to gender-affirming hormone treatment. Our study does not include a broader community-based sample of youth who do not or cannot access these centers and may have different patterns of mental health symptomology and potentially lower levels of gender affirmation. This highlights the need to reach youth either within their communities or within their primary care homes to determine if there are mental health and gender affirmation inequities not detected in our sample. Another possibility is that BLTY and WTY may have similar levels of depression symptoms, anxiety, and experiences of gender affirmation in adolescence, with disparities emerging during adulthood when risk factors for mental health symptoms—trauma, socioeconomic disadvantages, psychosocial stressors, and systemic discrimination—increase and psychosocial supports decrease [36]. Moreover, we did not measure other mental health symptoms such as post-traumatic stress disorder-related symptoms, which may be particularly salient to BLTY as trauma in the forms of victimization and race-based harassment are prevalent for these youth [13].

Also counter to hypotheses, BLTY reported lower rates of lifetime suicidality compared to WTY. Further investigation is needed to understand this finding with one potential confounder being culturally based stigma regarding suicidality which may affect self-reporting [37]. BLTY may also have more resilience against suicidality in the face of having to regularly navigate systemic racism and transphobia. Notably, 59% of BLTY endorsing lifetime suicidality is still a high prevalence rate and should be placed in the context of concerning national trends of Black and Latine youth populations experiencing substantial increases in suicide death rates compared to White youth [14,15]. These findings provide a critical foundation for understanding the experiences of all youth who experience gender minority and/or racial/ethnic minority-related stressors.

We sought to understand mental health symptoms in the context of gender affirmation and explore potential differences between BLTY and WTY. Consistent with our hypotheses, we found that higher levels of gender affirmation were associated with reduced odds of mental health symptoms in both subgroups. The literature has consistently documented the importance of gender affirmation in mitigating mental health risk among gender-diverse youth [25–27,29,38]. Our study uniquely demonstrates that these associations apply specifically to BLTY as well. Moreover, these findings could be powerful psychoeducational tools for caregivers and families of gender diverse youth, including BLTY, emphasizing the importance of both parental support and other forms gender affirmation. Our findings reinforce the importance of gender affirmation for gender-diverse youth in the United States who are facing increasing barriers to gender-affirming care and support. These barriers include politically driven assaults on the rights and gender affirmation of gender-diverse youth, including banning gender-affirming medical treatment for youth, preventing them from participating in sports, or even saying the word “transgender” in schools [39]. These barriers may be particularly salient for BLTY who are also simultaneously experiencing societal stigma and discrimination related to structural and system racism. To that end, our study provides evidence for how critical gender affirmation is for gender diverse youth, and especially BLTY, and the potential harmful repercussions if access to such support is absent or taken away.

The BLTY subgroup includes both Black and Latine youth, who undoubtedly have diverse and unique cultural experiences and realities. Our approach mirrors other studies that examine health and psychosocial outcomes in Black and Latine gender-diverse populations [3–5,13]. We focus on this subgroup as a crucial first step in documenting the experiences of potentially vulnerable youth who are marginalized at intersections of transphobia and racism and are presenting clinically for gender-affirming medical care. Future qualitative and quantitative studies are needed to explore the mental health impacts of intersectional stigma that individuals from specific racial/ethnic minoritized groups may experience. Future studies are also needed to understand culturally based factors that may influence BLTY’s access to various forms of gender affirmation. The willingness of parents of BLTY to affirm their child, support social transition, or pursue gender-affirming medical treatment may be influenced by culture-based gender norms, which shape gender-related beliefs and behaviors, including how someone responds to individuals who do not align with these gender norms [40]. Enlisting medical and mental health providers, community-based supports, and faith leaders to provide critical support to BLTY and their families in promoting gender affirmation could be an approach for culturally informed interventions and psychoeducation.

Our study has several additional limitations. Analyses were cross-sectional and, thus cannot infer causality among race/ethnicity, mental health symptoms, and gender affirmation. Additionally, generalizability is limited to youth presenting to pediatric subspecialty gender clinics in large cities. In particular, findings may not be generalizable to youth with limited English proficiency or have significant barriers to accessing gender-affirming medical services, including caregivers unsupportive of gender-affirming care, lack of insurance, and living in areas in which gender-affirming medical care is restricted or unavailable. Our findings may also have limited generalizability to gender-diverse youth who do not identify as transgender, which was the gender identity most represented in our study sample. In addition, the modest number of BLTY in our analytical sample limited our ability to compare mental health symptoms and gender affirmation between different races and ethnicities within the BLTY subgroup. Also, this may have limited the ability to fully detect BLTY and WTY subgroup differences in associations among mental health symptoms and gender affirmation measures. Another limitation of the study is that it did not measure other factors that may influence mental health symptoms or gender affirmation among BLTY, such as experiences of structural racism, racial/ethnic discrimination, intersectional stigma, or socioeconomic status; exploring such factors will be critical for future study. Additionally, the analyses presented here did not include other mental health outcomes but was limited to depression and anxiety symptoms and lifetime suicidality.

Our study, however, also possesses a number of strengths. Our study conducted novel comparisons between BLTY and their WTY peers. Additionally, the screening instruments used to assess mental health symptomology, perceived parent acceptance, and gender affirmation are considered empirically sound. Moreover, our study explored multiple forms of gender affirmation among BLTY and examined how these forms of gender affirmation were associated with baseline mental health symptoms.

In conclusion, our study’s novel focus on BLTY in comparison to the WTY peers allowed us to shed light on their experiences. Although it is an important step, it is not enough to explore race-and ethnicity-based differences among gender diverse youth, as race is only a proxy for the systemic stressors and discrimination that affect these youth. Future studies should focus on recruiting a more diverse sample of BLTY including youth outside of academic pediatric gender centers and include measures of structural racism and intersectional stigma to inform interventions to prevent and address health inequities among Black and Latine gender-diverse adults.

## Figures and Tables

**Table 1:** Demographics

Total cohort	Total (n = 262)	Designated female at birth (n = 177)	Designated male at birth (n = 85)	*p* value
Age; median (IQR)	16.0 (3.0)	16.0 (2.0)	17.0 (2.0)	<.001^b^
Gender identity				
Transmasculine	164 (63%)	164 (93%)	0 (0%)	
Transfeminine	81 (31%)	1 (1%)^a^	80 (94%)	
Nonbinary	17 (6%)	12 (6%)	5 (6%)	
Race/ethnicity group				.29
BLTY	92 (35%)	66 (37%)	26 (31%)	
WTY	170 (65%)	111 (63%)	59 (69%)	
Black and Latine transgender/nonbinary cohort	Total (n = 92)	Designated Female at Birth (n = 66)	Designated Male at Birth (n = 26)	*p*-value

Hispanic/Latine White	21 (23%)	19 (29%)	2 (8%)	.11
Hispanic/Latine Non-White	46 (50%)	30 (45%)	16 (61%)	
Black	9 (10%)	7 (11%)	2 (8%)	
Black and Hispanic/Latine	3 (3%)	3 (5%)	0 (0%)	
Black, Hispanic/Latine + other Races	1 (1%)	0 (0%)	1 (4%)	
Hispanic/Latine and other Races	7 (8%)	5 (7%)	2 (8%)	
Black & Other Races	5 (5%)	2 (3%)	3 (11%)	

BLTY = Black and Latine transgender/nonbinary youth; IQR = interquartile range; SD = standard deviation; WTY = White transgender/nonbinary youth.

a This participant reported gender identity at the baseline assessment as concordant with their designated sex at birth, but they did meet clinical criteria for gender dysphoria and were seeking medical gender affirmation.

b *p*-value <.05.

**Table 2:** Mental health symptoms for Black and Latine transgender/nonbinary youth and White transgender nonbinary youth

	BLTY	WTY	*p* value
Some degree of depression (mild/moderate/severe); n (%)	44 (48%)	95 (57%)	.19
Lifetime suicidality; n (%)	52 (58%)	120 (73%)	.02^a^
Clinically significant anxiety; n (%)	50 (57%)	101 (60%)	.59

Fisher exact test was used to compare dichotomous variables.

BLTY = Black and Latine transgender/nonbinary youth; WTY = White transgender/nonbinary youth.

a *p* value <.05.

**Table 3:** Experiences of gender affirmation for Black and Latine transgender/nonbinary youth and White transgender nonbinary youth

	BLTY	WTY	*p* value
	n(%)	n(%)	
Living full time as affirmed gender	74 (81%)	132(79%)	.75
	Median (IQR)	Median (IQR)	*p* value

Perceived parental acceptance; median (IQR)	24.0 (7.0)	24 (5.4)	.91
Non-affirmation; median (IQR)	17 (7.0)	16.9 (8.0)	.33
Internalized transphobia; median (IQR)	14 (13.0)	13.4 (13.0)	.88
Community connectedness; median (IQR)	13.0 (6.0)	14.0 (5.0)	.84

For dichotomous variables, Fisher exact tests were used. For continuous variables, independent sample *t* tests were used.

BLTY = Black and Latine transgender/nonbinary youth; IQR = interquartile range; WTY = White transgender/nonbinary youth.

**Table 4:** Association of gender affirmation with mental health symptoms

Mental health symptom	Primary predictor in separate models	Adjusted odds ratios^a^ (95% CI)	*p* value
Depression	Not living full time in affirmed gender	2.46 (1.21–4.88)	.012^b^
	Parental acceptance	0.91 (0.87–0.96)	.001^b^
	Non-affirmation	1.08 (1.03–1.13)	.002^b^
	Internalized transphobia	1.10 (1.07–1.14)	<.001^b^
	Community connectedness	0.91 (0.91–1.03)	.322
Lifetime suicidality	Not living full time in affirmed gender	1.38 (0.66–2.87)	.395
	Parental acceptance	0.96 (0.91–1.01)	.117
	Non-affirmation	1.10 (1.04–1.15)	<.001^b^
	Internalized transphobia	1.04 (1.01–1.08)	.009^b^
	Community connectedness	1.02 (0.96–1.09)	.498
Anxiety	Not living full time in affirmed gender	2.23 (1.07–4.63)	.031
	Parental acceptance	0.94 (0.89–0.99)	.022
	Non-affirmation	1.11 (1.05–1.17)	<.001^b^
	Internalized transphobia	1.10 (1.06–1.14)	<.001^b^
	Community connectedness	0.97 (0.91–1.03)	.317
	Interactions terms in separate models with gender affirmation X race/Ethnicity group	aOR	*p* value

Depression	Not living full time in affirmed gender x group	1.43 (0.35–5.87)	.616
	Parental acceptance x group	1.02 (0.92–1.13)	.688
	Non-affirmation x group	1.04 (0.94–1.14)	.460
	Internalized transphobia x group	1.05 (0.97–1.13)	.243
	Community connectedness x group	1.04 (0.91–1.19)	.540
Lifetime suicidality	Not living full time in affirmed gender x group	2.22 (0.49–10.0)	.301
	Parental acceptance x group	1.08 (0.97–1.20)	.166
	Non-affirmation x group	0.94 (0.85–1.04)	.264
	Internalized transphobia x group	1.00 (0.93–1.07)	.984
	Community connectedness x group	0.94 (0.82–1.08)	.351
Anxiety	Not living full time in affirmed gender x group	0.83 (0.19–3.54)	.798
	Parental acceptance x group	1.00 (0.90–1.11)	.973
	Non-affirmation x group	1.00 (0.91–1.11)	.886
	Internalized transphobia x group	1.04 (0.96–1.13)	.308
	Community connectedness x group	1.04 (0.90–1.20)	.579

aOR = adjusted odds ratio.

a All models are adjusted for age as a continuous variable and designated sex at birth.

b Indicates statistical significance at a false discovery rate of 0.05 after Benjamini-Hochberg procedure for multiple comparisons.
